# Integrating genome editing with omics, artificial intelligence, and advanced farming technologies to increase crop productivity

**DOI:** 10.1016/j.xplc.2025.101386

**Published:** 2025-05-28

**Authors:** Abigail Bradbury, Olivia Clapp, Anna-Sara Biacsi, Pallas Kuo, Oorbessy Gaju, Sadiye Hayta, Jian-Kang Zhu, Christophe Lambing

**Affiliations:** 1 Rothamsted Research, Harpenden, UK; 2 University of Lincoln, Lincoln, UK; 3 Department of Crop Genetics, John Innes Centre, Norwich Research Park, Norwich, Norfolk, UK; 4 Institute of Advanced Biotechnology, Southern University of Science and Technology, Shenzhen, China

**Keywords:** genome editing, robotics, artificial intelligence, farming, CRISPR, phenomics

## Abstract

Celebrated for boosting agricultural productivity and enhancing food security worldwide, the Green Revolution comprised some of the most significant advances in crop production in the 20th century. However, many recent studies have reported crop yield stagnation in certain regions of the world, raising concerns that yield gains are no longer sufficient to feed the exponentially growing global population. Here, we review the current challenges facing global crop production and discuss the potential of genome editing technologies to overcome yield stagnation, along with current legislative barriers that limit their application. We assess strategies for the integration of genome editing with omics, artificial intelligence, robotics, and advanced farming technologies to improve crop performance. To achieve real-world yield improvements, agricultural practices must also evolve. We discuss how precision farming approaches—including satellite technology, AI-driven decision support, and real-time monitoring—can support climate-resilient and sustainable agriculture. Going forward, it will be essential to address issues throughout the agricultural pipeline to fully integrate rapidly developing genome editing methods with other advanced technologies, enabling the industry to keep up with environmental changes and ensure future food security.

## Teaser

In the face of yield stagnation and the growing global population, integrating technologies such as robotics, artificial intelligence, and high-throughput omics and phenomics across the agricultural pipeline is essential to inform and implement advances in crop genetic improvement. This review examines key bottlenecks in developing new crop lines and the barriers limiting their translation into tangible yield gains in the field. In an era of rapid technological advances, increasing climate pressures, and shifting global policies, the strategies outlined in this review are critical for ensuring future food security.

## Introduction

**Figure 1:**
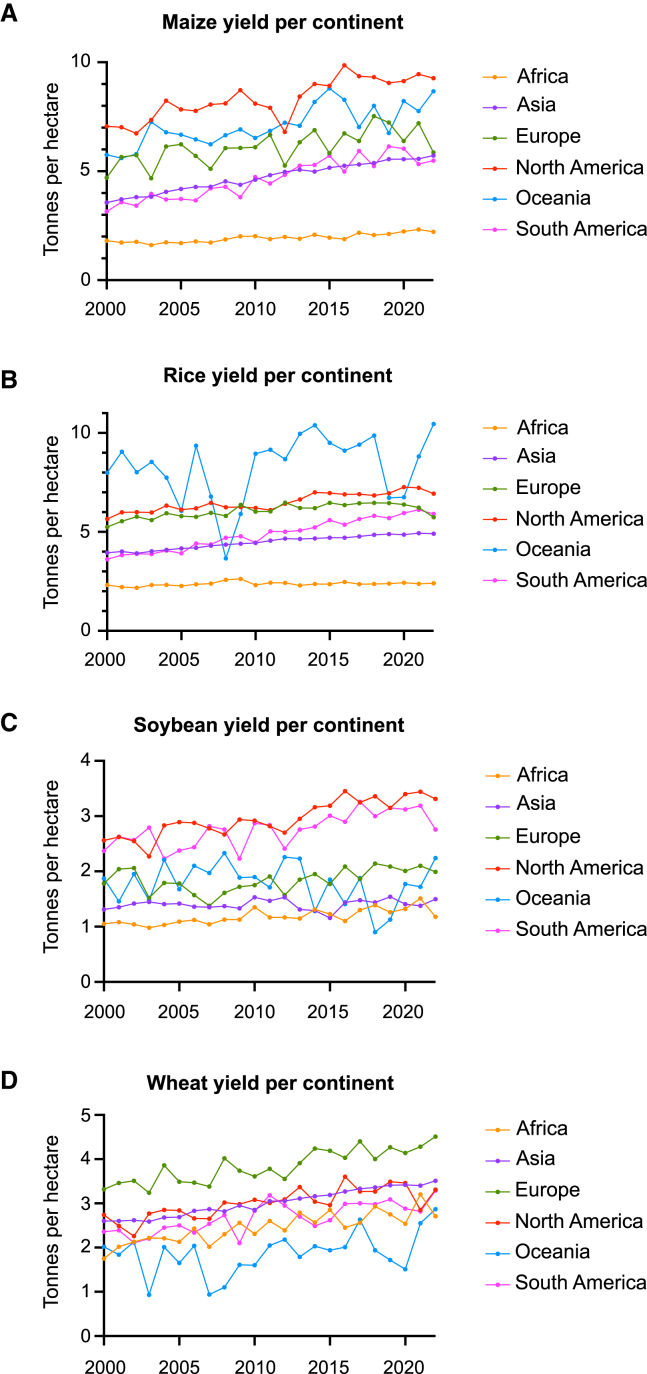
Yearly average yield (tonnes per hectare) by continent for wheat, rice, maize, and soybeans (2000–2022). **(A–D)(A)** maize yield, **(B)** rice yield, **(C)** soybean yield, **(D)** wheat yield. Yield data are from Ritchie et al., 2022.

From 1960 to 2000, agricultural productivity tripled due to the development and adoption of improved germplasms, combined with important advances in infrastructure and energy inputs (Evenson and Gollin, 2003; Briggs, 2009; Pingali, 2012). Although these techniques improved food security and prevented projected food shortages in many regions, they did not boost yields uniformly across all countries and crops (Figure 1) (Pimentel and Pimentel, 1990; Evenson and Gollin, 2003; Pingali, 2012; Liu et al., 2020b). Conventional breeding is slow, often requiring decades to generate new crop varieties, which limits its effectiveness in addressing urgent food security and environmental issues. Advanced techniques such as targeting induced local lesions in genomes (TILLING) and CRISPR-Cas-based mutagenesis enable precise genetic modifications and significantly accelerate the development of improved crop varieties. These innovations increase breeding efficiency and offer solutions to create resilient, high-yield crops more effectively than traditional methods. However, several bottlenecks continue to limit the application of genome editing in food production. This review gives an overview of the current challenges in crop production, discusses the limitations and potential of conventional crop breeding, and describes how genome editing technologies could address yield stagnation. It also evaluates current regulatory frameworks for gene-edited crops and proposes strategies for the integration of genome editing with other advanced technologies to improve the entire crop production pipeline and overcome yield stagnation.

## Current challenges in crop production

**Figure 2:**
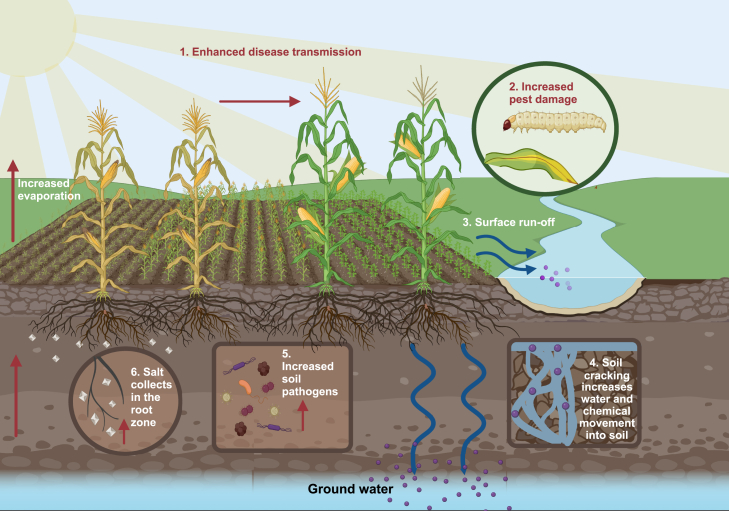
Effects of climate change on crops and the environment. Increased temperatures and growth of high-density monocultures accelerate disease transmission (1), pest damage (2), and soil pathogen density (5). The application of chemicals (purple circles) such as fertilizers and pesticides leads to their release into the environment (3). Global climate change causes soil cracking and increases chemical movement through the soil (4). Hot and arid climates increase soil salinization (white crystals represent salt) (6). Figure created with BioRender.

Crop genetic improvement and the use of pesticides, fertilizers, and irrigation have contributed significantly to yield gains but have also led to some unintended consequences for the environment and for long-term food production systems. Global pesticide production has increased by approximately 850% over the past 50 years (Pimentel and Pimentel, 1990; Grigg, 2001; Briggs, 2009; Pingali, 2012; McKenzie and Williams, 2015). However, pesticide use is remarkably inefficient: only about 1% of applied pesticides effectively control their target pests, and the rest enters the environment through leaching, adsorption, spray drift, and runoff, causing environmental damage (Figure 2) (Aktar et al., 2009; Tudi et al., 2021; European Environment Agency, 2023). Climate change will exacerbate the adverse effects of chemical pollution caused by high pesticide and fertilizer use. Rising temperatures increase soil erosion and cracking, which increase the movement of water and chemicals through the soil, risking surface and ground water contamination (Figure 2) (Tudi et al., 2021). In addition, increased irrigation used to support high yields has led to increased soil salinization in areas with poor drainage, which can lead to salt accumulation in the root zones of crops. This results in ion toxicity, nutrient imbalances, and reduced seed germination (Figure 2) (Briggs, 2009; Khamidov et al., 2022). As climate change alters temperature and precipitation patterns, soil salinization is expected to worsen in some regions, further reducing yields (Briggs, 2009; Jaggard et al., 2010; Tarmizi, 2019; Skendžić et al., 2021; Turin et al., 2023).

The introduction of monocropping replaced traditional intercropping practices that helped maintain rural biodiversity and encouraged pest resistance (Briggs, 2009). The growth of high-density monocultures enhances disease transmission among plants of high genetic homogeneity (Figure 2). This is exemplified by the breakdown of wheat resistance to stripe rust (Yr17) in England and Denmark, where cultivars containing a single resistance gene were grown over a wide area from 1994 to 1998, resulting in 100% virulence (de Vallavieille-Pope, 2004). High fertilizer use is also associated with increased levels of plant nutrients and soil minerals, which can increase the risk of disease and crop attractiveness to phytophagous pests (Pimentel and Pimentel, 1990; Grigg, 2001). In addition, climate change enables pest populations to expand into regions where they were previously absent (Skendžić et al., 2021). Crop pests and diseases are estimated to cause global yield losses of 21.5%, 30.3%, and 22.6% in wheat, rice, and maize, respectively, with plant pathogens costing the global economy an estimated $220 billion annually (He and Creasey Krainer, 2020; Ristaino et al., 2021).

Currently, many areas in the world are experiencing stagnation in yield growth, with many developing countries predicted to fall short of projected food demand due to insufficient yield increases (Figure 1) (Ray et al., 2013). Global average yields of maize, rice, wheat, and soybeans are increasing at annual rates of 1.6%, 1.0%, 0.9%, and 1.3%, respectively, far below the 2.4% annual increase required to meet projected demand (Ray et al., 2013). For example, in India, yield growth has stalled in some key production areas, with yield stagnation observed in 76% of wheat-, 47% of rice-, and 18% of maize-producing regions (George, 2014; Madhukar et al., 2020). This trend is particularly concerning in light of rising global undernourishment. Reversal of the decline in yield growth is vital to ensure sufficient food production in the coming years (World Health Organization, 2024).

## Limitations and prospects of traditional crop breeding

Crop breeding has been used to enhance the productivity of cultivated species through methods such as pure line selection, hybrid breeding, population breeding, pedigree breeding, and double haploid breeding. Despite its utility, breeding is becoming increasingly difficult due to dwindling genetic heterogeneity in cultivated varieties, a phenomenon known as genetic erosion (Khoury et al., 2022; Salgotra and Chauhan, 2023). An estimated 75% of plant genetic diversity has been lost over the past century (FAO). This loss is attributed to land use changes, climate change, and the replacement of local landraces with high-yield varieties (Khoury et al., 2022; Salgotra and Chauhan, 2023). Because plant genetic resources serve as important reservoirs of disease resistance and climate resilience genes, conservation of natural genetic variation for use in breeding programs is essential (Tanksley and McCouch, 1997; Bohra et al., 2022; Salgotra and Chauhan, 2023). Gene banks are the most widely used conservation method, with around 1750 gene banks storing approximately 7 million samples worldwide (FAO, 2010). Crop wild relatives are of particular conservation interest; they have not undergone the intense genetic bottlenecks associated with domestication and represent important sources of genetic diversity for trait improvement. However, they account for only 16% of gene bank holdings worldwide. Furthermore, although the introgression of genes from crop wild relatives is estimated to add $186 billion annually to the global economy, breeding efforts are often focused on members of the primary gene pool (close relatives) and overlook the greater benefits of crosses between more distantly related species (Tanksley and McCouch, 1997; Tyack et al., 2020; Bohra et al., 2022).

The introgression of improved traits into crop varieties is not always possible. Reproductive barriers between domesticated strains and their wild relatives can impede gene transfer between them and undesirable quality- and yield-related traits may also be introduced, thereby limiting the potential for improvement (Bohra et al., 2022). Desirable alleles can be transferred to progeny along with deleterious ones due to linkage drag, a phenomenon whereby two nearby loci remain genetically linked in the offspring population. These linked alleles are inherited together across generations, which presents an important challenge for conventional breeding methods, as the deleterious alleles are unlikely to be removed through crossing (Bohra et al., 2022). One potential strategy to overcome linkage drag is to engineer meiotic recombination by increasing the total number of recombination events and altering their genomic locations in germ cells. Recombination events occur during meiosis and can be modulated by temperature, epigenetic factors, or the overexpression or inactivation of genes that regulate meiotic recombination (Kuo et al., 2021; Fayos et al., 2022). Given the current limitations of conventional breeding and the average 7–12-year breeding pipeline required to generate a new line, conventional methods, although important, are unlikely to facilitate germplasm improvement quickly enough to address the rapidly changing climate and the exponentially growing global population.

## Development of genome editing techniques

**Figure 3:**
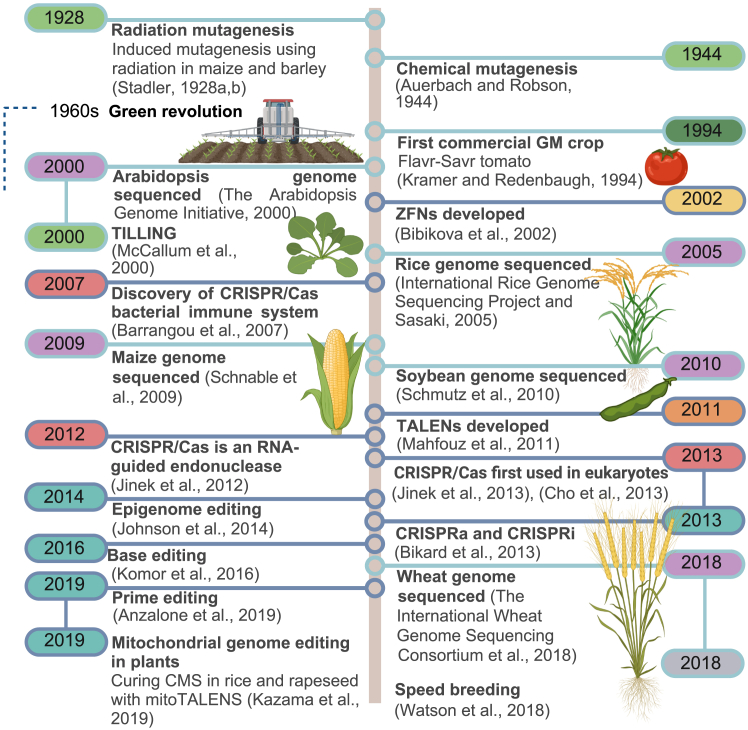
Timeline of milestones in crop genetic improvement. Light green: advances in mutagenesis; purple: sequencing of plant genomes; yellow: ZFN discoveries; red: CRISPR-Cas discoveries; orange: TALEN discoveries; light blue: expansions in precision breeding techniques. Genome editing technologies are indicated by dark blue lines (Arabidopsis Genome Initiative, 2000, Auerbach and Robson, 1944, Barrangou et al., 2007, Bibikova et al., 2002, Bikard et al., 2013, Cho et al., 2013, Jinek et al., 2012, Jinek et al., 2013, Johnson et al., 2014, Kramer and Redenbaugh, 1994, Mahfouz et al., 2011). Figure created with BioRender.

Since the first evidence of induced plant mutagenesis in 1928 using radiation in maize and barley (Stadler, 1928a, 1928b), scientists have used various approaches to create novel genetic variations and improve plant traits. The first mutant-derived varieties emerged in the late 1950s and early 1960s, including *Golden Promise* barley and canola varieties of oilseed rape (Figure 3) (Shelake et al., 2019). In 2000, TILLING was introduced as a technique that combines traditional crossbreeding, chemical mutagenesis, and DNA analysis to induce desired mutations and generate new lines (Figure 3) (McCallum et al., 2000). The original TILLING protocol, a relatively short-lived method for screening mutant populations, has since been largely superseded by genomic methods with broader applicability such as EcoTILLING (Comai et al., 2004), iTILLING (Bush and Krysan, 2010), De-TILLING (Li et al., 2001), and PolyTILLING (Wang et al., 2012). These methods facilitate the creation and identification of new alleles in both coding and non-coding regions and are applicable to large genomes, enabling the creation of mutant populations suitable for direct use in breeding programs (Singh et al., 2024). Successful applications of TILLING-based approaches for crop improvement include the development of oilseed rape with improved oil quality (Wang et al., 2008; Lee et al., 2018) and tomato lines resistant to *Potato virus Y* and *Pepper mottle virus* (Piron et al., 2010).

**Figure 4:**
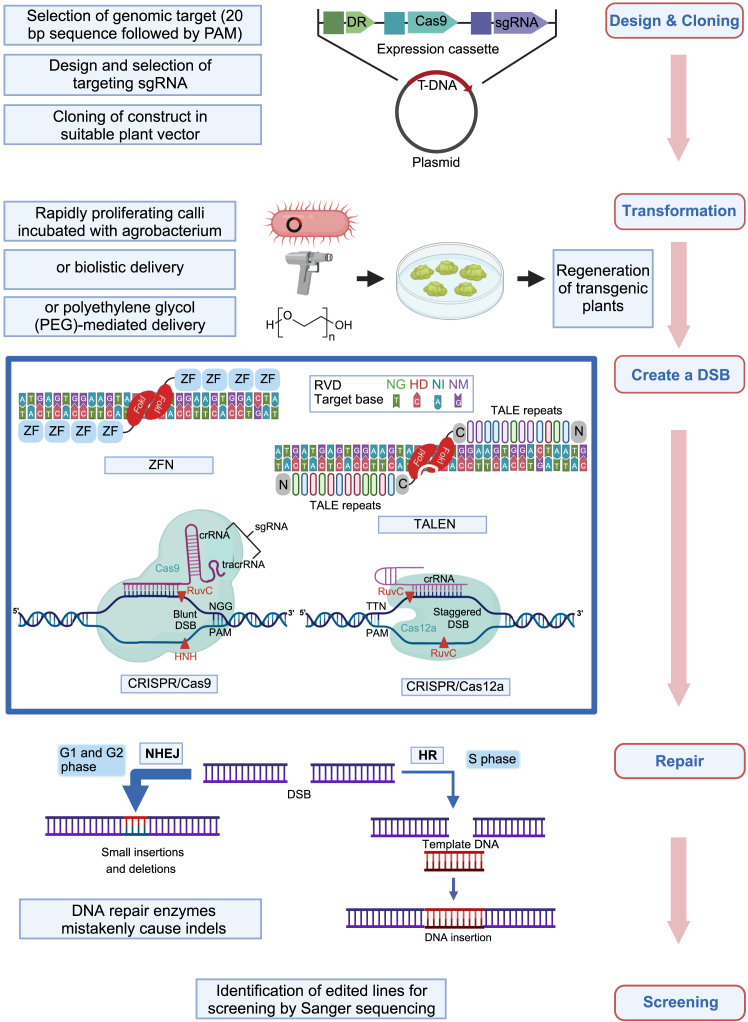
General genome editing pathway. Schematic representation of genome editing procedures, including design and cloning, plant transformation, DSB formation, DSB repair, and screening of transformed plants. The design and cloning phase shows a plasmid containing a developmental regulator (DR) gene, Cas9, and a single guide RNA (sgRNA). Calli (green) are transformed via *Agrobacterium* (red), or protoplasts (green) via biolistic delivery (gene gun, gray) or polyethylene glycol (PEG). NGTs are shown in the blue box as mechanisms to engineer targeted DSBs. ZFN: A pair of zinc finger proteins, each with four DNA-binding domains (blue boxes) and a C-terminal FokI nuclease (red), joined by a spacer (black line). TALEN: Two transcription activator-like effector (TALE) proteins with effectors. Each effector has a repeat variable di-residue (RVD) that binds to a specific nucleotide (shown in the light blue box). Each TALE protein is linked to a C-terminal FokI nuclease (red) by a spacer. CRISPR-Cas9: An sgRNA (purple) is bound to DNA (dark blue) at its target site next to the PAM sequence “NGG.” Cas9 (cyan) uses its RuvC and HNH domains to cut the DNA at two sites (red triangles) on opposite strands. CRISPR-Cas12a: The guide RNA only consists of crRNA (not tracrRNA). The PAM sequence “TTN” is located at the 5′ end of the DNA. The cleavage sites (red triangles) are offset, which creates a staggered DSB. All DSBs may be repaired by the NHEJ pathway, which can introduce small insertions or deletions, or by the HR pathway, which can introduce DNA insertions. Figure created with BioRender.

Despite these successes, the randomness of DNA mutagenesis results in high levels of unwanted background mutations that need to be removed through multiple rounds of backcrossing. Chemical and radiation mutagenesis cannot be used for rapid genome engineering, which led to the development of targeted mutagenesis systems using endonucleases that induce double-strand breaks (DSBs) at specific genomic sites. These DSBs are repaired by endogenous mechanisms, typically by error-prone non-homologous end joining (NHEJ), which introduces insertions or deletions at the repair site (Figure 4). In some cases, homologous recombination (HR) is used, which allows precise edits via donor DNA templates with homology arms (Figure 4). However, HR is used less frequently, as it is limited to somatic S-phase and meiosis, whereas NHEJ is active throughout most of the somatic cell cycle (Symington and Gautier, 2011). The first targeted mutagenesis system was based on zinc finger nucleases (ZFNs), which consist of a DNA-binding domain from a zinc finger transcription factor fused to the non-specific DNA cleavage domain of the Type IIS restriction enzyme FokI (Figure 3). A major limitation of this system is the difficulty of predicting the DNA-binding sites of the zinc finger domains (Khalil, 2020), and it took 9 years from the discovery of ZFNs to their first application in plant genome editing (Townsend et al., 2009). In 2009, the discovery of transcription activator-like effectors (TALEs) in the phytopathogen *Xanthomonas oryzae* led to the development of a new system based on TALE-nuclease fusions (TALENs) that generate DSBs in a manner similar to ZFNs (Figures 3 and 4). TALEs are simpler to design, as each module recognizes a single nucleotide, resulting in binding sites that are significantly more predictable than those of ZFNs and therefore reduced off-target effects. However, the construction of TALEs can be labor-intensive (Supplemental Table 1) (reviewed in Khalil, 2020; Zhang et al., 2018).

In 2012, the development of the clustered regularly interspaced short palindromic repeats (CRISPR)-Cas system revolutionized genetic engineering by enabling precise and efficient genome editing (Figure 3). Derived from a viral defense mechanism originally discovered in bacteria, CRISPR-Cas9 technology uses a single guide RNA (sgRNA), a fusion of a CRISPR RNA (crRNA) and a trans-activating CRISPR RNA (tracrRNA), which directs the Cas9 nuclease to a target DNA sequence. This enables efficient, precise gene editing by matching the 5ʹ crRNA base-pairing region with the complementary target sequence (Figure 4) (reviewed in Gao, 2021). Over the past few years, CRISPR-Cas9 has dominated the genome editing field, significantly advancing plant research and offering great potential for crop improvement (Li et al., 2021b). It is a versatile, simple, and inexpensive tool for sequence-specific DNA modification, including gene knockout, single-base substitution, gene or allele replacement, and multiplex genome engineering (Cong et al., 2013; Mali et al., 2013; Li et al., 2021b) (Supplemental Table 1). When multiple sgRNAs are used to induce multiple DSBs, the system can cause chromosomal deletions, gene inversions, and chromosomal translocations, and can target multiple genes simultaneously (Sedeek et al., 2019; Beying et al., 2020; Lu et al., 2021; Rönspies et al., 2022). Many novel Cas orthologs with additional advantages have been identified; for example, Cas12j has a shorter coding sequence that facilitates vector delivery (Sun et al., 2024), Cas12a has a different PAM recognition sequence (Zhang et al., 2023), and Cas13 targets RNA viruses (Hak et al., 2024; Kavuri et al., 2022). The engineering of Cas proteins represents a novel avenue for the expansion of the genome editing toolbox. For example, Cas-SF01 is an artificial intelligence (AI)-guided, genetically engineered derivative of Cas12i3 with enhanced gene editing activity in both animals and plants (Duan et al., 2024).

**Figure 5:**
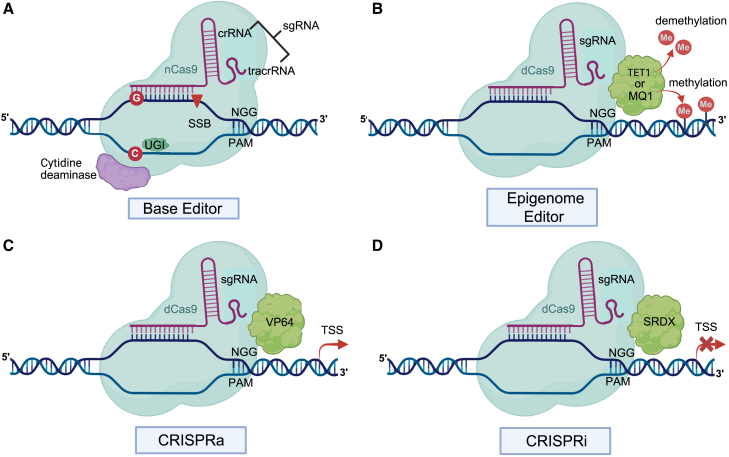
Expanding genome editing technologies. **(A)** Schematic representation of a cytosine base editing system (CBE): nCas9 (cyan) is fused to a cytidine deaminase (purple), which catalyzes the conversion of cytosine (red circle) to uridine. A uracil glycosylase inhibitor (UGI) prevents the U:G mismatch from reverting to C:G, resulting in a change to T:A. The sgRNA, which is made up of a CRISPR RNA (crRNA) and a trans-activating CRISPR RNA (tracrRNA), guides nCas9 (cyan) to the target site. Upon recognition of the PAM motif “NGG,” nCas9 introduces a single-strand break (SSB, red triangle), which is processed by the base editor. **(B)** Schematic representation of an epigenome editing system: dCas9 (cyan) is fused to either the TET1 or MQ1 epieffector domain, which catalyzes DNA demethylation and methylation, respectively. The sgRNA and PAM sequence direct dCas9 to the target site. **(C)** Schematic representation of a CRISPR activation (CRISPRa) system: dCas9 is fused to the transcriptional activator VP64. “TSS” denotes the transcription start site. **(D)** Schematic representation of a CRISPR interference (CRISPRi) system: dCas9 is fused to the transcriptional repressor SRDX. Figure created with BioRender.

The applications of genome editing technologies in breeding are rapidly expanding (Table 1) (reviewed in Zhu et al., 2020). Base editing, developed in 2016, enables the direct conversion of one target DNA base to another without requiring DSB formation or a donor template (Gaudelli et al., 2017; Komor et al., 2016; Nishida et al., 2016; reviewed in Li et al., 2021b; Molla et al., 2021). This method involves the fusion of a cytidine deaminase enzyme with an engineered CRISPR-Cas9 that lacks nuclease activity (CRISPR-dCas9) but is still targeted to a specific DNA sequence by its guide RNA (Figure 5A). The first successful applications of this method in crops were demonstrated in wheat, rice, tomato, and maize (Lu and Zhu, 2017; Li et al., 2017; Ren et al., 2017; Shimatani et al., 2017; Zong et al., 2017). Because base editing is limited to specific nucleotide substitutions, new methods with broader editing capabilities have been developed. Prime editing, described in 2019, is a “search-and-replace” genome editing system capable of targeted insertions, deletions, and all 12 types of base-to-base substitutions (Anzalone et al., 2019; reviewed in Li et al., 2021b; Molla et al., 2021). It consists of a reverse transcriptase fused to an RNA-programmable nickase and a prime editing guide RNA (pegRNA). The genetic information from the pegRNA is copied directly into the target locus, enabling greater versatility and precision than base editing (Anzalone et al., 2019). Although prime editing technology has low editing efficiency in plants, improved systems have been developed to overcome this limitation (Li et al., 2022a; Jin et al., 2023; Ni et al., 2023). For example, prime editors were used to insert a 30-base pair (bp) *cis*-regulatory element into the promoter of the rice *R* gene *Xa23* to confer resistance to bacterial blight (Gupta et al., 2023). Although prime editing can achieve targeted insertion of short *cis*-regulatory elements, the insertion length is limited and multiplexing is difficult. Lu et al. (2020) developed an efficient method for inserting both short and long sequences at target sites in the plant genome. This method involves particle bombardment of callus cells with CRISPR-Cas constructs to generate DSBs at target sites and chemically modified double-stranded donor DNA fragments that bear 5ʹ-phosphorylation and both 5ʹ and 3ʹ phosphorothioate linkages on each strand. The modified donor DNA is stable in cells and can be inserted efficiently at the DSB sites. For example, the insertion of four TALE-binding elements into the promoters of the rice executor genes *Xa10* and *Xa23* conferred resistance to all tested *Xanthomonas oryzae pv. oryzae (Xoo)* strains (Zhang et al., 2024b).

**Table 1:** Applications of genome editing toolkits for crop improvement.

Crop	Target gene	Genome editing	Trait improvement
Strawberry	*FaPG1*	Mutagenesis (CRISPR-Cas9)	Improved fruit firmness (López-Casado et al., 2023)
Soybean	*AIP2a, AIP2b*	Mutagenesis (CRISPR-Cas9)	Increased protein content (Shen et al., 2022)
Wheat	*TaGW2*	Mutagenesis (CRISPR-Cas9)	Increased yield (Wang et al., 2018)
Tomato	*SlWUS, SlCLV3, SlWOX9, SlTFL1*	Mutagenesis (CRISPR-Cas9)	Variation in fruit size, inflorescence branching, and plant architecture (Rodríguez-Leal et al., 2017)
Maize	*ARGOS8*	Mutagenesis (CRISPR-Cas9)	Increased drought tolerance (Shi et al., 2017)
Soybean	*FAD2-1A, FAD2-1B, FAD3A*	Mutagenesis (TALEN)	High oleic acid content (Demorest et al., 2016)
Rice	*Os11N3*	Mutagenesis (TALEN)	Increased bacterial blight resistance (Li et al., 2012)
Maize	*IPK1*	Mutagenesis (ZFNs)	Herbicide tolerance and reduced phytate levels (Shukla et al., 2009)
Wheat	*ALS*	Base editing (CRISPR-based)	Herbicide resistance (Zhang et al., 2019)
Strawberry	*FvebZIPs1.1*	Base editing (CRISPR-based)	Fine-tuned sugar content (Xing et al., 2020)
Maize	*ZmALS1, ZmALS2*	Base editing (CRISPR-based)	Herbicide resistance (Li et al., 2020c)
Rice	*Xa5, Xa23*	Prime editing	Increased bacterial blight resistance (Gupta et al., 2023)
Rice, rapeseed	*ORF79, ORF125*	Mitochondrial gene mutagenesis (mitoTALENs)	Cytoplasmic male sterility (Kazama et al., 2019)
Lettuce	*psaA, psbA, rrn16*	Base editing of the chloroplast genome	Herbicide resistance (Mok et al., 2022)
Cassava	*MeSWEET10α*	Epigenome editing	Increased bacterial blight resistance (Veley et al., 2023)

Mitochondrial and chloroplast genome editing have great potential for improvement of the respiratory and photosynthetic pathways through crop breeding; however, they require specific modifications of technologies currently used for nuclear genome editing (Dorogova and Sidorchuk, 2023). The primary challenge is the apparent absence of the NHEJ repair pathway in these organelles. Instead, the HR pathway is active, reflecting their prokaryotic origin, which limits the introduction of mutations via DSB induction and repair (Maliga, 2022). CRISPR-Cas9 also faces challenges because sgRNA is difficult to transport across the mitochondrial membrane, a limitation not seen with TAL effectors (Supplemental Table 1). The first successful application of this approach was the use of TALENs fused to N-terminal mitochondrial localization signals (mitoTALENs) to knock out genes associated with cytoplasmic male sterility (CMS) in rice and rapeseed (Kazama et al., 2019). Base editing has also been applied using TALEs fused to nucleotide deaminases (TALEDs), enabling the introduction of point mutations in mitochondrial and chloroplast genomes. DddAtox-derived cytosine base editors (DdCBEs) are highly effective TALEDs constructed by the fusion of TALEs with the DddAtox cytidine deaminase domain (Li et al., 2021a). DdCBE-mediated editing was first implemented in mitochondria and has recently been adapted for chloroplasts (Zhang et al., 2024a; Kim and Chen, 2024). This approach has been effective in engineering herbicide resistance in lettuce and creating a stop codon in the rice chloroplast gene *psaA* (Li et al., 2021a; Mok et al., 2022).

Epigenome editing represents another avenue for crop improvement. CRISPR-dCas9 can methylate or demethylate cytosines at a target site, thereby modulating gene expression levels (Qi et al., 2023). In plants, CRISPR-dCas9-mediated DNA methylation has recently been developed using a variant of the bacterial CG-specific DNA methyltransferase MQ1 (Figure 5B). MQ1 has reduced activity but high specificity, enabling accurately targeted *de novo* DNA methylation in *Arabidopsis* (Ghoshal et al., 2021). Targeted methylation in the CG context induces phenotypic changes in plants that can be maintained through mitosis and meiosis without introducing genetic mutations. Similarly, CRISPR-dCas9 fused with the catalytic domain of the human demethylase TEN-ELEVEN TRANSLOCATION1 (TET1cd) has been used for targeted DNA demethylation in *Arabidopsis* (Li et al., 2020b). The dCas9-SunTag transcriptional activator system has also been adapted for site-specific DNA methylation editing in plants. Fusion of TET1cd with the dCas9-SunTag system allowed targeted demethylation and activated gene expression of the well-characterized *FWA* epiallele in *Arabidopsis* (Figure 5B) (Gallego-Bartolomé et al., 2018). This system has also been successfully used to change DNA methylation and gene expression, and to create epialleles that are heritable to the next generation in rice (Tang et al., 2022). In another study, the tobacco methyltransferase catalytic domain NtDRMcd was used with the SunTag system to methylate the *FWA* promoter and induce early flowering (Papikian et al., 2019). Epigenome editing has also been used to increase bacterial blight resistance in cassava (Veley et al., 2023). Given its potential, further exploration of epigenome editing for crop breeding is warranted.

Overall, genome engineering techniques such as TILLING and CRISPR-Cas-based systems enable precise genetic modifications and unlock valuable genetic traits that might otherwise remain inaccessible. These tools expand genetic diversity and provide breeders with new opportunities to develop resilient, high-yield crops.

## Bottlenecks in the delivery of genome editing components into plants

Since the advent of CRISPR-Cas9 genome editing, efforts to refine and eliminate bottlenecks in the process have been made to enable global implementation of the technology in support of food systems. A major bottleneck that limits the full potential of genome editing in crop breeding is the delivery of genome editing reagents, as Cas proteins are large and delivery mechanisms must be species-specific (Atia et al., 2024). In vegetatively propagated crops such as potato, targeted gene mutations have been achieved through transient expression of CRISPR-Cas9 ribonucleoproteins in protoplasts (Andersson et al., 2017; Tuncel et al., 2019). Similarly, delivery of preassembled CRISPR-Cas9 ribonucleoproteins into lettuce protoplasts has produced transgene-free mutant plants (Woo et al., 2015). However, the regeneration of plants from cultured protoplasts remains very challenging for most monocotyledons, particularly major cereal crops. Tissue culture-free strategies such as RNA virus-mediated transformation, nanoparticles, and polyethylene glycol (PEG)-mediated delivery have also been used; however, these face their own challenges, including cell damage, cargo size limitations, and low efficiency in plant cells (Figure 4) (Wang et al., 2022b; Cardi et al., 2023; Hwarari et al., 2024).

One of the most widely used methods to transfer genetic material into plants is *Agrobacterium*-mediated transformation, which involves infection of the plant with an engineered *Agrobacterium tumefaciens* strain. sgRNA and Cas can be expressed either transiently or from a transgene integrated into the plant genome as part of a T-DNA construct (Zhang et al., 2016). This method has some limitations, including low transformation efficiency and a restriction to plant species susceptible to *A. tumefaciens* infection. To improve this method, T-DNA vectors are increasingly designed to include developmental regulator genes (DRs) that induce embryogenesis or organogenesis from somatic cells in tissue culture and promote the growth of transformed plants (Nasti and Voytas, 2021). DR expression is particularly advantageous in plant species that are recalcitrant to regeneration or have long regeneration times (Laforest and Nadakuduti, 2022). DRs such as PGA37/MYB118 (Wang et al., 2009), WUS2, BBM (Lowe et al., 2016), STM (Maher et al., 2020), and WOX5 (Wang et al., 2022a) have demonstrated regeneration-promoting effects in plant transformation. However, constitutive DR expression can cause negative pleiotropic effects and infertility, necessitating their removal from transgenic plants and limiting their practical utility. As an alternative, the expression of a growth-regulating factor (GRF) and GRF-interacting factor (GIF) as a GRF4-GIF chimera has been shown to increase the speed and efficiency of plant regeneration (Debernardi et al., 2020). Co-delivery of the GRF4-GIF chimera with CRISPR-Cas9 on the same T-DNA vector enhances regeneration efficiency in both monocotyledonous and dicotyledonous species, resulting in fertile edited plants (Debernardi et al., 2020). An important approach to overcome the plant regeneration bottleneck is to integrate rapid genome editing directly into speed breeding systems that use optimized light intensity, temperature, and photoperiod control, combined with an early seed harvest to reduce generation times (Watson et al., 2018; Hussain et al., 2023). In approaches such as ExpressEDIT, Cas9–sgRNA constructs are directly introduced into plants, and rapid trait selection is used to identify plants that lack Cas9 but carry the desired trait and segregate them from plants that retain Cas9 and can undergo further editing cycles (Hickey et al., 2019).

## Global policies on genome-edited crops

The emergence of new genome engineering technologies presents opportunities to develop crops with improved agricultural values. Given the potential of genome engineering tools, it is surprising that 166 of 195 United Nations-recognized countries prohibit genetically modified organisms (GMOs). It is often observed that neighboring countries have similar stands on the use of genome-edited crops and GMOs, with countries in the Americas and Asia having less stringent regulations than Africa and Europe. Given the potential of genome editing to increase yield gains, and the fact that about 1 in 11 people globally suffer from hunger, the prohibition of genome editing in plants needs further examination. Africa’s population is projected to reach 2.5 billion by 2050, and food production in the region will need to increase to prevent the exacerbation of pre-existing food insecurity (United Nations Department of Economic and Social Affairs, 2017). For many major crops grown in Africa, realized yields fall well below potential yields. For example, maize is a staple crop in sub-Saharan Africa, but the average grain yield in Africa is 2.1 tons/ha/year, much lower than the global average of 5.8 tons/ha/year (Woomer et al., 2024). This yield gap is also underpinned by abiotic and biotic stresses. Although genome editing has the potential to reduce the yield gaps of several staple African crops, only four African countries have regulatory policies that permit genome-edited crops. This is despite the African Union’s 2023 strategic framework stating in 2023 that one of their aims was to improve productivity and crop disease resistance through the use of genome editing (Buchholzer and Frommer, 2023). In 2020, Nigeria became the first African country to implement guidelines that permit genome-edited crops (Report of the House Committee on Environment and Habitat, 2020), followed by Kenya and Malawi in 2022, and Ghana in 2023 (Ledford, 2024). Several other African countries are currently considering regulatory policies for genome editing, including Burkina Faso, South Africa, Ethiopia, Sudan, Eswatini, and Zimbabwe (Tripathi et al., 2022).

The international regulatory environment for genetic technologies is evolving rapidly, and an increasing number of countries are revising their policies to exclude genome-edited crops from existing GMO regulations. Argentina became the first country to make such a change in 2015, establishing what is now known as the “Argentina model.” This model exempts genome-edited plants that contain no permanent insertion of foreign DNA, with regulatory decisions made on a case-by-case basis (Whelan and Lema, 2015). Several other countries subsequently passed similar legislation, including Chile (2017), Brazil (2018), Colombia (2018), and the United States (2018) (Buchholzer and Frommer, 2023; Zarate et al., 2023). The United States, like Argentina, regulates GMOs based on the genetic composition of a plant rather than the method used to engineer it, whereas the EU’s Court of Justice ruled in 2018 that organisms developed using new genomic techniques (NGTs), including genome-edited crops, remain subject to stringent GMO regulations. However, the EU has since drafted new regulations to revise the risk assessment process for NGT-derived plants (Watson and Hayta, 2024). Countries such as Japan, Canada, the United States, and Argentina have adopted proportionate regulatory systems for precision breeding, in which targeted genetic changes are approved if they could have arisen naturally or through conventional breeding. In China, genome-edited crops that do not contain foreign DNA still require risk assessment before regulatory approval, although the process is less stringent than that used for GMOs (Zhu, 2022). The UK, after it left the EU in 2020, reconsidered its stance on genome-edited crops; in 2022, the UK government introduced a statutory instrument to amend the existing GMO regulations. In addition, under the Genetic Technology (Precision Breeding) Act 2023, plants and animals developed through precision breeding technologies were excluded from GMO regulatory requirements and became subject to more proportionate and less restrictive regulations. However, this legislation only applies in England, as the devolved governments of the UK have all rejected it to date. In the near future, it is likely that more countries will re-examine their regulatory systems for genome-edited crops as public understanding of genome editing technologies improves and the effects of climate change on crop yield become harder to mitigate.

## Combining genomics and phenomics to inform genome engineering strategies

**Figure 6:**
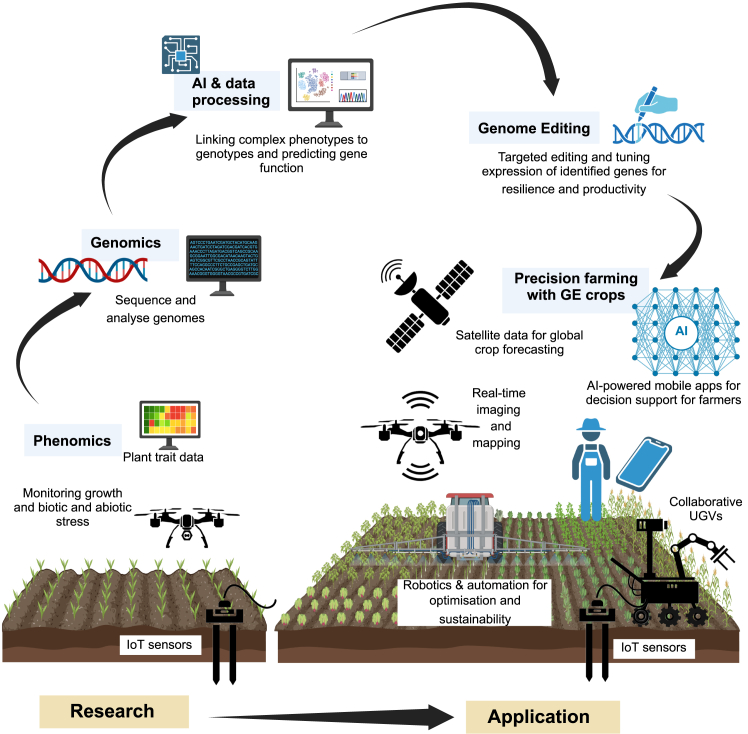
Integration of genome editing and advanced technologies to increase crop productivity. Overview of how advanced technologies can be translated from research to real-world agricultural application. Phenomics and genomics enable the identification of target genes, informing genome editing strategies that develop resilient, high-yield crop varieties. In the field, precision farming approaches involving robotics, AI, IoT networks, and satellite imagery result in optimized resource use, reduced yield gaps, and expanded productivity in less arable regions. Figure created with BioRender.

With the availability of affordable and efficient genome editing tools and the implementation of less stringent regulations on genome-edited crops, attention is shifting toward the identification of target genes for editing. For instance, yield is a highly complex polygenic trait that is difficult to noticeably improve by targeting a single gene (Cao et al., 2020). Moreover, plant breeders constantly aim to improve both yield and stress resistance, traits that are often antagonistic. A 20-year project by Corteva Agriscience assessed the effects of 1671 genes on yield, nitrogen use efficiency, and drought tolerance in maize and identified 22 genes with relevant physiological functions (Simmons et al., 2021). Genetic redundancy in polyploid species such as wheat poses another challenge, as it can obscure novel alleles associated with improved agronomic traits. In view of these challenges, a holistic approach that combines genetics, metabolomics, genomics, phenomics, and environmental data is required to identify genes and regulatory pathways underlying complex traits and to predict crop performance under variable climatic conditions (Figure 6). This approach successfully provides extensive knowledge to support the design of precise crop improvement strategies. This is supported by a recent multi-omics study that sequenced the genomes of 1035 wheat varieties, including both Watkins landraces and modern cultivars, and collected 717 000 phenotypic observations across 137 traits; the study identified 8253 genetic effects, including 15 novel loci conferring resistance to yellow rust (Cheng et al., 2024).

High-quality reference genomes are essential resources for omics approaches and studies of gene function (Adamski et al., 2020; Yao et al., 2025). The genomes of rice, maize, soybeans, and wheat have been sequenced, with their annotations released in 2005, 2009, 2010, and 2018, respectively (Figure 3) (International Rice Genome Sequencing Project and Sasaki, 2005; Schmutz et al., 2010; Schnable et al., 2009; International Wheat Genome Sequencing Consortium et al., 2018). Despite their utility for scientists and breeders, these genomes contain gaps composed of unknown sequences, along with sequences that cannot be assigned to specific chromosomes because of insufficient sequence continuity. Long-read DNA sequencing is a powerful gap-filling technique for genome assemblies (Liu et al., 2020a; Aury et al., 2022; Chen et al., 2023). As the technology improves, it is being used to characterize natural genetic and structural variation across large accession sets to generate extensive genomic data that support the identification of agriculturally relevant loci and guide future breeding programs (Li et al., 2020a; Shang et al., 2022; Zhang et al., 2022).

Plant phenomics is not a new concept; Furbank (2009) described plant phenomics approaches as a means to provide the quantitative phenotyping required to determine the genetic basis of agricultural traits and to screen germplasm for genetic variation. Many countries have invested in plant phenomics platforms for the analysis of canopy and root traits under controlled and field conditions. Platforms may be ground-based or aerial (using manned or unmanned aerial vehicles) and can be manually operated, vehicle-mounted, or robotic. Several institutes and universities have invested heavily in the development of phenotyping platforms designed for either controlled environments (Sadok et al., 2007) or field conditions (Virlet et al., 2016). In field trials, phenotypic data are collected by drones equipped with RGB cameras to measure crop growth rates and/or thermal cameras to create field maps and detect biotic and abiotic stresses such as pests, diseases, and drought. Many institutions have also developed data integration and storage systems for crop phenotypic data. Two notable systems are: (1) the Internet of Things (IoT)-based CropSight platform, an open-source information management system for automated data acquisition by sensors and phenotyping platforms; and (2) the Phenotyping Hybrid Information System, developed by the French National Institute of Agricultural Sciences ( INRA), which integrates and manages phenotypic data from multiple experiments and platforms using an ontology-driven architecture. These platforms are an extensive data resource that supports gene discovery based on traits.

A vast amount of data that needs to be processed has been generated. Robotics and autonomous systems have emerged as next-generation technologies with considerable potential to transform agricultural practices (Pearson et al., 2022). The phenomics approach holds promise for translating gene discovery to farmgate (Furbank, 2009), but the “big data” challenge of how to process the massive datasets generated by sensors on phenotyping platforms remains a major bottleneck. AI has emerged as an essential tool to address this problem, with the potential to sustain and boost agricultural output. AI is being adopted across almost all spheres of life. It can collect, manage, and process large numbers of datasets from multiple omics experiments and climatic data to precisely link complex phenotypes with genotypes and to predict gene function and crop performance (Figure 6) (Khan et al., 2022). Crop traits such as plant height and leaf area can be measured with high accuracy using AI-driven sensors and imaging systems, enabling rapid screening of breeding lines (Benos et al., 2021). Machine learning and deep learning approaches have shown great potential in extracting image-based phenotypic data (Khan et al., 2022; Poorter et al., 2023). Other promising AI models include DeepBind and DeepSEA, which analyze genetic features; DeepBSA, which maps genetic regions linked to phenotypic variation (i.e., quantitative trait loci); and AlphaFold, which uses deep learning to predict protein structures (Alipanahi et al., 2015; Zhou and Troyanskaya, 2015; Jumper et al., 2021; Li et al., 2022b). These tools enable myriad possibilities that can advance omics research by accelerating the identification of genes relevant to crop breeding.

## Use of robotics and AI to maximize the agricultural output of genome-edited crops

Maximizing the agronomic benefits of genome-edited crops requires precision farming (also referred to as smart farming) approaches that leverage robotics, AI, and the IoT to improve sustainability and maximize yields (Figure 6) (Sharma et al., 2023). These systems provide farmers with real-time information on crop health and soil conditions, supporting field-specific evaluations and informed decision-making on irrigation, pesticide use, and fertilization to maximize agricultural output (Figure 6). Uncertain weather conditions make it very hard to predict crop performance. IoT networks connect sensors, drones, and data-processing systems to monitor the climate, soil conditions, and crop health. IoT sensors placed in fields collect data on soil moisture, acidity, and nutrient content; combined with aerial imagery and environmental data, these inputs allow AI models to predict stress factors and optimize irrigation, fertilization, and pesticide use (Sharma et al., 2023). AI-driven thermal imaging analysis can rapidly detect nutrient deficiencies, allowing timely corrective action before yield loss occurs.

AI-powered decision support systems and mobile applications are further transforming farm management (Figure 6). These tools provide real-time updates on pest outbreaks, disease progression, and weather patterns, allowing farmers to respond proactively. Mobile phone based applications have proven especially useful in bridging knowledge gaps, particularly in regions with limited access to other information and communication technologies, such as computers (Ayim et al., 2022). Recent advances include deep learning models for the early detection of diseases, such as mango leaf disease, and integrated platforms that combine real-time crop diagnostics with e-commerce services, weather information and government market updates (Aslam et al., 2024; Puranik et al., 2024). These technologies empower smallholder farmers and help reduce global yield gaps by expanding access to important precision farming insights.

A recent development in agricultural monitoring is the NASA–ISRO Synthetic Aperture Radar (NISAR) satellite, scheduled for launch in 2025. NISAR’s dual-frequency radar can penetrate clouds and crop canopies, providing high-resolution, uninterrupted global crop monitoring twice every 12 days (ICO SSR, 2025). This capability will allow farmers and policymakers to monitor crop growth, soil moisture, and biomass levels in real time; optimize planting schedules, irrigation, and resource allocation; and enhance global crop forecasting and food security planning. Public access to these data and integration with AI-driven decision support systems and mobile applications could further transform farm management, particularly in regions with limited access to monitoring technologies such as sensors and drones (Jet Propulsion Laboratory, 2025).

Precision and smart farming also integrate AI with unmanned ground vehicles (UGVs) and robotic systems for automated planting, monitoring, and harvesting (Figure 6). As climate change drives agriculture into new environments and genome-edited crops resilient to more extreme conditions are developed, robotics will be crucial in enabling the cultivation and management of these crops in locations other than the traditional flat fields (Botta et al., 2022). Platforms such as AgriQ address the challenges posed by uneven terrain, confined areas, and poor global positioning system (GPS) reception (Botta and Cavallone, 2021). Collaborative UGVs and drones equipped with multispectral sensors can map fields, monitor crop growth, and optimize resource allocation. Autonomous weeding robots from companies such as ecoRobotix use AI to identify weeds and selectively apply herbicides with 6 × 6 cm precision, reducing herbicide usage (Bykov, 2023). Similarly, robotic harvesters increase efficiency for labor-intensive crops like strawberries (Chang and Huang, 2024) and tomatoes (Kim et al., 2022), minimizing post-harvest losses. Using these robotic systems with genome-edited crops can further enhance productivity, ensuring that agricultural practices keep up with advances in plant science and produce crops for a growing population in the context of climate change. Adoption of precision and smart farming practices along with genome editing technology could alleviate yield stagnation, enhance product quality, and reduce environmental footprint, delivering significant social, economic, and environmental benefits.

## Concluding remarks and perspectives

Genome editing technologies are a powerful tool to introduce new traits into crops and improve agricultural productivity. Their applications are rapidly expanding, from editing of single-bases edits to long nucleotide sequence insertions, and their scope continues to grow as new Cas orthologs with distinct PAM specificities are developed. Genome editing targets are no longer limited to the nuclear genome; mitochondrial and chloroplast genome editing enable access to previously inaccessible photosynthetic and respiratory genes. Emerging epigenomic editing techniques allow trait improvements without altering the genome and enable control of transcriptional regulation to induce nuanced changes in gene expression levels. This less permanent editing approach may face fewer regulatory constraints and holds potential for broader implementation.

To maximize the impact on crop production, genome editing should be integrated with complementary innovations such as speed breeding, phenomics, AI, robotics, and satellite technologies (Figure 6). Although regulatory restrictions on the commercialization of genome-edited crops remain a challenge, a growing number of countries are exempting such crops from these regulations, facilitating broader agricultural adoption. This relaxation of regulations, combined with new technological advances, could support the development of crop varieties suited to address the challenges caused by climate change. Meaningful progress will require not only technological innovation but also a cohesive pipeline that involves collaboration among biotechnologists, agronomists, engineers, plant breeders, farmers, agribusinesses, and policymakers. Increased communication across these sectors will be essential to translating advances in genome editing and AI-driven technologies into practical agricultural solutions that address global yield stagnation, food security, and climate resilience.

## Funding

This work was supported by the 10.13039/501100001809National Natural Science Foundation of China grant 32188102 to J.-K.Z. and by the 10.13039/501100000268Biotechnology and Biological Sciences Research Council grant BB/X011003/1 to C.L.

## Acknowledgments

We would like to thank Nigel Halford for his comments on the manuscript. No conflict of interest declared.

## Supplemental information

Document S1. Supplemental Table 1

Document S2. Article plus supplemental information
