# Supplementary material for: Integrating genome editing with omics, artificial intelligence, and advanced farming technologies to increase crop productivity
Source: Plant Commun. 2025 May 28;6(7):101386. doi: 10.1016/j.xplc.2025.101386 (PMC12281252; doi:10.1016/j.xplc.2025.101386)
Supplement: Document S1. Supplemental Table 1 [file mmc1.pdf]

Plant Communications, Volume *6*

Supplemental information

Integrating genome editing with omics, artificial intelligence, and advanced farming technologies to increase crop productivity

Abigail Bradbury, Olivia Clapp, Anna-Sara Biacsi, Pallas Kuo, Oorbessy Gaju, Sadiye Hayta, Jian-Kang Zhu, and Christophe Lambing

Supplementary Information

Table S1. Summary of the genome editing toolkits with their advantages and limitations.

	**ZFNs**	**TALENs**	**CRISPR-Cas**
**Type of recognition**	Protein-DNA interaction	Protein-DNA interaction	RNA-DNA interaction
**Nuclease**	Fok I	Fok I	Cas9 and its variants and orthologues
**Prediction on-target / off-target binding sites**	Difficult	Feasible	Highly feasible
**Engineering**	Difficult.<br>Need to engineer a new protein for each target site	Moderate.<br>Complicated cloning approach due to the repeated motifs	Highly feasible.<br>Synthesise a new gRNA for each new target site.
**Multiplexing**	Difficult	Difficult	Highly feasible
**Applications with and without nuclease activity**	Mutagenesis<br>Epigenome editing	Mutagenesis<br>Base editing<br>Epigenome editing<br>Mitochondrial/chloroplast genome editing	Mutagenesis<br>Base editing<br>Prime editing<br>Epigenome editing
